# A new approach for explosion accident prevention in chemical research laboratories at universities

**DOI:** 10.1038/s41598-022-07099-2

**Published:** 2022-02-24

**Authors:** Koji Fukuoka, Masao Furusho

**Affiliations:** 1 Risk Management Office, Kyushu University, 744 Motooka, Nishi-ku, Fukuoka, 819-0395 Japan; 2 President Office, National Institute of Technology, Oshima College, Oshima-gun, Japan

**Keywords:** Health occupations, Chemistry, Engineering

## Abstract

Over the years, many accidents have occurred during chemical experiments in laboratories around the world. However, the methods of investigating and analysing accidents that have occurred at universities have not been consolidated, and the lessons learned from these accidents have not been shared. In this study, accident investigation reports of explosions in chemistry laboratories at two universities were analysed with an analysis tool based on the software/hardware/environment/liveware (SHEL) model. As a result, university accidents were classified as epidemiological models, and it became clear that the contributing factors to the accidents, which were investigated and analysed using the SHEL model, can be used as learning experiences and therefore applied for the prevention of accidents at other universities. Universities around the world need to come together to formulate research and analysis methods, rules for creating accident reports, etc. and provide a place for sharing information that will enable them to make use of the lessons learned from all kinds of accidents.

## Introduction

An accident that occurs during a chemical experiment at a laboratory in a university could kill faculty members, researchers, graduate students or students^1,2^. While it is unclear how many accidents have occurred in laboratories at universities around the world, such accidents have subsequently reoccurred at other universities, and in these cases, no paradigm shift or drastic changes have occurred regarding laboratory safety^3^. Additionally, no progress has been made in terms of the universities collaborating to prevent these accidents.

From 2009 to 2016, the author worked as an accident investigator for the Japan Transport Safety Board of the Ministry of Land, Infrastructure, Transport and Tourism of Japan, conducting investigations and analyses of many ship accidents in Japan and around the world and creating accident investigation reports. Afterwards, he was involved in safety-related work as an emergency response coordinator and manager of the Occupational Health and Safety Section at the Okinawa Institute of Science and Technology Graduate University (OIST). However, he came to learn about accidents occurring at universities across the world, which have killed numerous faculty members and students; 126 accidents^4^ related to chemical substances occurred in laboratories in educational and research institutions, including universities in the United States, between January 2001 and July 2018. In one case, a research assistant, Sheharbano Sangji, died after suffering burns in a laboratory at the University of California, Los Angeles, in 2008^5^.

In terms of accident prevention, the difference between universities and maritime/aviation fields is that international rules pertain to the accident investigations of aircraft and ships, and international organizations, such as the International Civil Aviation Organization (ICAO) and International Maritime Organization (IMO), stipulate accident investigations and analysis methods and their rules, gather data and disseminate the learnings obtained from the data globally^6,7^. In the field of aviation, the accident rate of commercial aircraft has been decreasing because of accident investigation and analysis and the accompanying safety recommendations^8,9^.

One university does not have investigation and analysis methods and tools, while another university uses the 4Ms (man, machine, media and method) as an analysis tool, which is applied in the manufacturing industry. No unified rules pertain to accident investigation and analysis or investigation reports of accidents that occur at universities, and the lessons learned from these accidents are not published; additionally, no international organizations work on accident prevention. According to Hollnagel, it is important for accident investigation and analysis to determine the accident model that applies to the concerned industry and carry out investigation and analysis by methods that fall under that accident model^10^. Accident models are classified into three types, namely, sequential models, epidemiological models and systemic models, and the classification depends on whether there is a link between the cause and effect, the characteristics of the accident occurrence mechanism and other factors^11^. Typical accident models include the domino model, Swiss cheese model^12^, and systems-theoretic accident model and processes^13^. In accident investigations, the use of different accident models will lead to the adoption of different mechanisms that lead to the occurrence of accidents, and the analysis and safety recommendations derived from them will deviate from the aim of preventing the recurrence of accidents and thus be ineffective^10,14^.

The mechanism of the sequential model is that an accident is a chain of unexpected events and circumstances, starting from a root cause and evolving linearly to an accident, and that an accident can only be prevented by removing one of the linearly arranged domino blocks^10^. This model cannot be applied to accidents involving many accident factors at the same time, such as explosions in chemical research laboratories, because this model is characterised by a chain of events, which makes it impossible to take the analysis one step further. The mechanism of the epidemiological model is that an accident is the result of a combination of an active failure and many latent conditions that were put in place directly and indirectly by humans and existed before the accident occurred and that the way to prevent accidents is to rectify the active failure and the latent conditions. The SHEL model is developed to cover all the human factors associated with accidents^15^. Using the SHEL model at the scene of an accident allows investigators to collect a comprehensive set of accident-related evidence at the investigation stage, leading to active failure and many latent conditions, and to draw safety measures derived from this investigation and analysis^16^. The mechanism of the systemic model is that an accident is caused by an imbalance in the components of a system without a cause-and-effect link and human errors because of the complexity of the whole system due to the multiple functions of a single component, which makes it impossible for people to predict an accident. This model applies to the nuclear industry and requires continuous monitoring of the operation of the system^10^.

Chemical substance-associated accidents that have occurred in university laboratories have been reported as case studies on some occasions^17^; however, no discussions have occurred regarding which of the three accident models they belong to. Therefore, no unified tool is available for accident investigation and analysis, and in addition to the contributing factors that were derived from the accident investigation, the data results that were derived when statistically processing data of the contributing factors were also questionable.

In this study, two accident investigation reports concerning accidental explosions during a chemical experiment in the laboratories of Texas Tech University^18^ and the University of Hawaii at Manoa^19,20^ were analysed using the accident investigation tool stipulated by the aforementioned international organizations, and the contributing factors were clarified. From the analysis results, university accidents can be classified into epidemiological models, and the contributing factors obtained from the analysis results can be considered learning experiences that can be used to prevent accidents. For several universities to benefit from the lessons learned, universities around the world must come together and formulate rules for the method of investigation and analysis, report making, etc., conduct education and training on the investigation and analysis methods and set up a place for sharing information to prevent accidents.

## Methods

In the case study of universities, we selected accident investigation reports that describe local workplace factors and organizational factors^12^. When performing an independent investigation of a university department in which an accident occurred, a proper investigation of the organizational factors of the university may not be possible because of the interests of the organizational structure. To eliminate this negative effect, a third-party expert joined the investigation team and selected a report that was investigated and analysed.

During the initial stages of the accident investigation, the circumstances leading to the occurrence of the accident were schematized in chronological order; however^21^, this schematic was omitted since the purpose of this study was to extract the contributing factors and prevent accidents.

This analysis used the SHEL model, which is used in aircraft accidents. Since the environment in which aircraft are operated differs from the environment of a university laboratory, it is not appropriate to use the details of each element for the same model, as drawn by Hawkins, because it is for accident investigation at a university. We used the same method^22^ for which this model was applied to ship accidents and modified the subdivisions as follows. (1) S stands for software; it is the procedure and rules used at the accident site. (2) H is the hardware; it includes the machines/equipment/facility’s condition and the human–machine interface. (3) E denotes the environment, which includes meteorological and oceanographic conditions, environmental conditions of the workplace, traffic movement in experimental sites and geographical characteristics. (4) Lc is the Central Liveware and indicates the cause of the accident of the operator. Lc is further divided into physical/sensory limits, physiological situations, psychological limits, individual workload management, knowledge/skills/experience/education and training. (5) Lp is the Peripheral Liveware located around Lc and is classified into the following aspects: communication between the experiment participants, teamwork, near-miss response, experiment discontinuation criteria, the operational status of the safety management system, the university environment, and involvement of external organizations that affect the safety of the university.

Teamwork indicates that the roles and responsibilities of the persons at the site are clear^23^. A near-miss response includes the means of addressing near-misses that occurred before the accident. The lessons learned from the near-miss analysis are almost the same as the lessons learned from accident analysis, the mechanism has been clarified, cases of saving lives using the lessons learned have been published, and it is an indispensable element for preventing accidents^14^. Experimental discontinuation criteria are the judgement of whether to restart an experiment or not. For example, the safety-related document may have to describe the criteria for discontinuing an experiment on chemical substances and such circumstances in the event of a near miss. The actual operation of the safety management system shows the formulation and compliance of various regulation manuals, implementation of university-wide education and training, evaluation of the participants’ abilities and systematic education and training programmes, improvement of defects that were found during safety inspections, etc. The university environment involves safety awareness and management initiatives, professors, researchers, etc. and problems of the organization structure from the viewpoint of ensuring the safety of the university. The involvement of external organizations that affect the safety of the university refers to the actual state of safety requirements concerning government agencies and private organizations.

In the analysis procedure for this study, the details described in each accident investigation report were classified according to each element and subdivision of the aforementioned SHEL model, and the causes of accidents at two universities were listed. The details given in the accident investigation report are plotted; therefore, the items not given in the report are not described.

This article does not contain any studies involving human participants performed by any of the authors.

## Results

The direct cause of both accidents is shown in Table 1, and the local workplace factors and organizational factors shown in Tables 2 and 3 have been clarified according to each SHEL element classification. Although these two accidents differed in type, there was a link between cause and effect in both cases, and there were several common factors. The summary is as follows. (1) In the S procedure, standard operating procedures were lacking, and personal protective equipment was not worn. (2) In terms of the psychological limit of Lc, the researchers did not possess a high level of awareness of the risks posed by the chemical substances being handled. (3) In terms of Lp communication, there was insufficient communication between the researchers and principal investigator. Regarding the near-miss response, a near miss occurred before the accident in the same laboratory; however, it was not investigated and analysed, and thus, the accident could not be prevented. In the safety management system, laboratory inspections conducted by inspectors of the health and safety department were not effective, and in Case Study 2, laboratory-specific substances were not inspected. The inspections were ineffective because the inspection is considered a violation of academic freedom when it is conducted in the absence of the principal investigator or the researcher, and thus, cooperative aspects between the principal investigator and researcher were lacking. Although the chemical hygiene plan was an important safety guideline, it did not describe the risk assessment techniques as a tool for assessing hazards. Research funding agencies did not demand university laboratory-specific safety regulations, risk analysis, etc.

**Table 1:** Types and causes of accidents.

	Case Study 1 (Texas Tech University)	Case Study 2 (University of Hawaii)
Type of accident	Explosion of chemical substances	Explosion of inflammable mixed gas
Cause of accident	The student used more than the prescribed usage limit of 100 mg of nickel hydrazine perchlorate (NHP); therefore, the powder exploded while the NHP was being stirred with a pestle	An electrostatic discharge occurred when a researcher used a pressure gauge, which ignited the hydrogen/oxygen gas mixture in the gas storage tank in which the explosive gas mixture was stored at high pressure, resulting in an explosion

**Table 2:** Local workplace factors.

SHEL Element	Subdivision	Case Study 1	Case Study 2
Software	Procedure at the accident site	There was no protocol or standard operating procedure for synthesising high-energy substances, and wearing personal protective equipment was left to the individual’s discretion	There was no protocol or standard operating procedure for working with explosive gas mixtures, and there was no usual practise of wearing personal protective equipment
Hardware	Condition of equipment		There was no grounding or bonding. A tank for dry air was being used
Environment	Environment of workplace, such as a laboratory		There was enough humidity to generate static electricity
Central liveware	Psychological limits	Based on experience, it was found that a small amount of compound does not ignite or explode on impact even when it gets wet with water or hexane, and the risk of a large amount of NHP was thought to be similar	Work dealing with high-risk substances and processes were not always recognized as high risk by many researchers
	Knowledge, skill, experience, education and training	Learning of techniques and methods associated with high-energy substances was completed with a literary review only; general laboratory safety training and education and training specific to this study were not undertaken	There was a lack of awareness that a mixture of inflammable gases and oxidant gases could ignite due to an electrostatic discharge or metal friction when an incubator or bioreactor is opened
	Communication	It was at the student’s discretion regarding when to consult the principal investigator for experiment or scale-up changes. There was no related governing university policy	The researcher told the principal investigator that the thesis had discussions about the need for keeping the O_2_ level within 4.0–6.9% and asked the principal investigator whether this factor should be considered and whether a fireproof lab coat was required, but (research institute) the principal investigator’s response was not known
		There were two communication tools for group meetings and research notes, but they were mainly focussed on experimental results and not used for safety purposes

**Table 3:** Organizational factors.

SHEL Element	Subdivision	Case Study 1	Case Study 2
Peripheral liveware	Near-miss response	Two near misses occurred in the same research group three years before the accident. The first time, nitrogen was generated just before the completion of the reaction product and an explosion was heard. The second time, the students made a mistake when scaling up and set the measurement unit to 30 g. These near misses were not documented as lessons learned and were not disseminated to the entire university	A day before the accident, electrostatic sparks were observed several times in ungrounded metal equipment, e.g., a “cracked sound” within the 1-gallon pressure vessel when the same digital gauge’s on/off button was pressed; however, the cause was not investigated and analysed, thereby losing the opportunity to prevent the accident
	Experiment discontinuation criteria		If a high-risk situation is observed in the event of a near miss, all work, including that with high-risk chemical substances and processes, must be discontinued, and all procedures must be investigated thoroughly
	Operational status of the safety management system	The environmental health and safety inspector had conducted a safety audit/inspection of 118 chemical laboratories prior to the accident, but the principal investigator did not take corrective action in many of the cases	The laboratory safety inspection conducted with the environmental health and safety office’s “Laboratory Safety Inspection Checklist” was not comprehensive and was reduced to a mere formality. The use of gas storage tanks was not confirmed, and it was necessary to have a section dedicated to compressed gas
		The chemical hygiene plan did not include comprehensive risk assessment guidance for the laboratories	The chemical hygiene plan did not elaborate on how researchers should best handle safety regulations and practices
		There was no obligation to undergo laboratory safety training that was provided online and in-person by the environmental health and safety staff. There was also video training offered for undergraduate students, but the need for hazard assessment before starting research in the laboratory was not mentioned	There was no policy or procedure to ensure that laboratory-specific safety trainings for individual laboratories are conducted regularly
		There was no documented procedure and approval process for any changes to experimental plans	Some laboratories lacked or did not have standard operating procedures for handling hazardous substances, implementing hazardous work or having protective barriers or emergency procedures
	University environment	The inspection was reduced to a mere formality and carried out in the absence of the principal investigator because the principal investigator considered it a violation of academic freedom	Lab safety inspection lacked cooperative aspects, such as inspecting the laboratory in the absence of the researcher
		The environmental health and safety office was stipulated to supervise chemical hygiene, but it was not under the authority of the vice president for research, and there was no authority to close the laboratory	Many researchers underestimated the chemical, biological and physical hazards and the need for personal protective equipment
	Involvement of external organizations affecting the safety of the university	The laboratory standards of the Occupational Safety and Health Administration did not address the physical hazards	Funding agencies did not demand risk analysis of studies that used explosive substances
		Funding agencies did not have a policy to limit the quantity of high-energy compounds that could be synthesized and lacked guidelines for assessing the risks in the laboratory	

## Discussion

First, let us discuss the contributing factors to these accidents. Schröder et al. conducted a comparative study of safety awareness and practices in the laboratories of universities (n = 991), government agencies (n = 133) and industries (n = 120). The rate of wearing personal protective equipment was lower in university laboratories than in laboratories of other institutions, and the more researchers recognized that the risk of their experiments was low, the lower the likelihood of wearing personal protective equipment was^24^. In Case Study 1 and Case Study 2, personal protective equipment was not worn at the time of the accident, and no standard operating procedures were in place. Personal protective equipment was not worn because it was not required in Case Study 1 and because the risks were underestimated or not properly understood in Case Study 2. On the other hand, in Case Study 2, the UC Centre for Laboratory Safety, which was the agency investigating the accident in the same report stated that, “*Once trained, the hazard often becomes a routine part of their experimentation, and researchers perceive themselves to be experts in handling the hazard. Perceived familiarity can shift the awareness level from cautiousness to complacency*”^19^. It has been noted that complacency, which refers to not recognizing a hazard properly due to becoming accustomed to it, is one human factor that is also relevant in other industrial fields^15^. One of the methods for tackling complacency is to always check assumptions against facts^23^. In the case of laboratory safety, it is necessary to prepare experimental protocols^24^ and guidelines and conduct education and training so that there is proper awareness of the hazards specific for each research study and the corresponding risk assessment can be carried out appropriately.

Schroder et al. showed that few accidents are correlated with involving principal investigators or supervisors^24^. The report described that in Case Study 1, there was no clear policy stating when the researcher/student is to consult with the principal investigator, and in Case Study 2, the researcher had asked questions of the principal investigator but received unclear answers. In an investigation conducted by the American Chemical Society on university experiments, there were no data from researchers; however, 70.5% of faculty members and 52.1% of graduate students conducted experiments individually rather than in groups^5^. From the investigation and this case study, it appears that there is a problem with the method of involvement of principal investigators or supervisors, because an external agent notices the mistake^25^. Therefore, if there is a mistake in an experiment that is conducted alone by an individual, the individual proceeds with the experiment without noticing the mistake, which increases the accident risk. Thus, avoiding experiments conducted individually by researchers/university graduates/students can help prevent accidents.

The relationship between principal investigators, supervisors and inspectors can be considered in terms of how they view academic research and safety regulations; 16% of university researchers said that safety rules affect productivity, and 26% said that safety rules interfere with the process of scientific discovery^24^. Some university researchers have a negative view of safety rules, inferring that safety and research activities diverge and are not integrated under some circumstances. Given that one of the purposes is to obtain experimental results while maintaining the safety of the laboratory, in both case studies, it was found that information sharing between the principal investigator and inspectors was not conducted well and that an authority gradient seemed to exist between the inspector and principal investigator. When there is an authority gradient within a team that shares the same purpose, useful information will not be shared, which will lead to accidents^15,23^. For workplace inspections to be collaborative and effective, it is necessary to clarify the responsibilities and authorities of inspectors and principal investigators, share the safety information and experimental information held by both parties and discuss where safety issues are present so that experiments can be conducted safely. Achieving experimental results and ensuring safety are not heterogeneous or separate but rather integrated, and risk assessment has to be an inseparable element of an experiment, as suggested by the National Research Council^26^.

In terms of experiment discontinuation criteria for preventing accidents, risk management and safety assurance processes suggest that risk assessment should be conducted again when the control is not effective or when there are new hazards^9^. In some case studies, some standards must be set for discontinuing an experiment when a hazardous situation is encountered. As noted by the accident investigation agency of Case Study 2, if the laboratory patrol or near-miss investigation learns that the safety of the laboratory is in imminent danger if the experiment is continued, it is necessary to grant authority, including the power to discontinue the experiment, to the agency in which the inspector belongs and investigate and analyse the near-miss.

These are targeted and specific accident prevention measures in terms of software, hardware, environment, and central liveware based on the accident analysis content of the SHEL model. Regarding software, the handling of chemicals used in experiments should be carried out under experimental protocols, standard operating procedures and other documents that describe the safe conduct of experiments. Regarding hardware, the equipment used should be suitable for the intended experiment and be properly grounded, and maintenance and inspection of the equipment should be carried out regularly. Regarding the environment, the atmosphere in the laboratory should not be conducive to the combustion or explosion of specific chemicals and should be monitored continuously. Regarding central liveware, because some researchers tend to become accustomed to hazards in the laboratory and do not recognize hazards properly, experiments conducted alone should be avoided, guidelines for hazard recognition and risk assessment specific for each research study should be developed, and education and training should be provided. In addition, group meetings and research notes should be used as a forum for discussion of safety-related issues and shared among laboratory members, faculty and departments.

Second, we will study the significance of the contributing factors that were obtained in the analysis during this study.

Investigation and analysis of ship accidents have revealed that the same types of accidents occur due to almost the same contributing factors, and in the case of different types of accidents, the contributing factors also differ to some extent^22^. In each of the universities in which the accidents of Case Study 1 and Case Study 2 occurred, all the contributing factors shown in Tables 2 and 3 must be corrected to prevent the recurrence of similar types of accidents. By applying these accident prevention measures at these universities and correcting these factors, latent defects can be eliminated, and recurrence can be prevented. On the other hand, regarding the lessons learned from this case study, other universities can compare the contributing factors that were extracted in this study with similar contributing factors potentially present at their university. Taking measures according to the results of this investigation can also reduce the risk of occurrence of the same types of accidents. In this study, the explosion of chemical substances and the explosion of an inflammable mixed gas were analysed, and each accident was expressed as a different event; however, the analysis of the contributing factors of each accident showed that some factors were common. In other words, the risk of two types of accidents can be gradually decreased by correcting the common factors.

The pattern of occurrence of accidents can be clarified if many accidents of the same type are recorded, investigated, and analysed and if common contributing factors are extracted^22^. Recurrence prevention measures for each type of accident utilizing these contributing factors will be useful for safer experimental protocols or the revision of health and safety plans and standard operating procedures. In addition, incorporating these measures as a new type of education and training will help increase the safety awareness of principal investigators, researchers and students and effectively contribute to preventing accidents.

Third, we will describe an accident model that can be applied to university accidents. Regarding the contributing factors, Tables 2 and 3 show that many contributing factors were present in both case studies. In other words, the analysis results of the two case studies showed that they did not belong to the sequential model, in which accidents occur with linear contributing factors, but instead belong to the epidemiological model, in which the mechanism of accident occurrence is due to several contributing factors. The systemic model assumes that there is no link between the cause and result of the accident; therefore^10^, the application of this model is excluded. According to Perrow, the university belongs to the 4th quadrant when classifying each industry by interaction and coupling^27^. Hollnagel and Speziali evolved it into an accident model classification to which Perrow’s classification can be applied. According to the model, universities were assigned to the 4th quadrant, and it was unclear what kind of accident model would be applied^28^. As a result of this study, it was found that the epidemiological model can be applied to university accidents. Determining the accident model classification has significance for applying it to various types of accidents that have occurred at universities.

**Figure 1:**
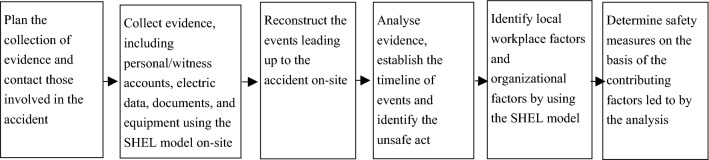
Flow chart on how an accident should be investigated. Adapted from Ref.^30^, International Maritime Organization.

Fourth, we will describe the method for investigating and analysing university accidents. The epidemiological model includes the Swiss Cheese model, SHEL and Reason hybrid model, human factor analysis and classification, and the Australian Transport Safety Bureau (ATSB) model^14^. As an accident investigation and analysis tool, the SHEL model has already been established as a scientific method and is used not only in the field of aircraft accidents but also for ship accidents^29,30^. The SHEL model-based investigation and analysis method that was used in this study can be applied to accidents that have occurred in university laboratories. Additionally, at OIST, where the author has worked previously, the department in charge of health and safety had been investigating and analysing accidents by incorporating a SHEL model-based analysis method into the accident investigation guidelines^31^. Figure 1 is a flow chart showing how an accident should be investigated. To eliminate the safety measures governed by the application and misapplication of common sense^32^, it would be beneficial for many universities to adopt comprehensive accident investigations and analyses using the same model to show evidence-based contributing factors and prevent accidents using the lessons learned.

Safety standards for university chemistry experiments are detailed in *Guidelines for Chemical Laboratory Safety in Academic Institutions*, which lists (1) recognize the hazards, (2) assess the risks of the hazards, (3) minimize the risks of the hazards, and (4) prepare for emergencies from uncontrolled hazards. The guidelines state that laboratory accident near misses should be reported and shared and that accident investigation and reporting are important for accident prevention^33^; however, they do not clarify the details. This study provides details and clarification on the significance and methods of accident investigation, including near misses and the use of contributing factors and lessons learned for accident prevention.

## Limitations of the study

The following two points can be considered limitations of this study. In the two case studies selected for this study, it was unclear what kind of investigation tools were used by the accident investigation agency when carrying out the investigation and analysis on-site. If a SHEL model-based investigation and analysis were shown by this study to have been conducted, it would have facilitated a comprehensive investigation covering subdivisions of all contributing factors. However, if the same model had not been used, some of the factors given in the subdivisions of the SHEL model may have existed at the time of the accident, and despite that, the accident investigator may have overlooked these factors during the investigation stage and not mentioned them in the accident investigation report. In Case Studies 1 and 2, subdivisions of the SHEL model were not investigated, such as the human–machine interface, physiological situations and individual workload management. If the SHEL model were to be used to collect evidence on-site, all subdivisions would have been investigated and analysed. Therefore, the analyses in Tables 2 and 3 are different from the actual situation at the time of the accident, and it may not be possible to prevent a recurrence of accidents even after correcting all the contributing factors described.

There are only two samples in this study—the causes of an explosion when handling chemical substances and the causes of an explosion of inflammable mixed gas—which are characteristic of this case study only and may not be considered common contributing factors for each type of accident. Therefore, for the lessons learned from accidents at other universities to be more effective at preventing accidents, it is necessary to gather many accidents of the same type for statistical significance and to carry out the investigation and analysis using the method applied herein to identify common contributing factors.

## Conclusions

In this study, we showed that an epidemiological model can be applied to accidents that have occurred in university laboratories and that a tool based on the SHEL model can be used as a method for investigation and analysis. The study also provided a method for preventing accidents using contributing factors identified via analysis. To reduce accidents, many universities must use the lessons learned from the analysis of all types of accidents. To do so, the accident investigation has to be scientific, the investigation and analysis methods have to be unified, and data from many accidents need to be gathered and analysed. For this approach, universities around the world must come together to formulate rules that include investigation and analysis methods and report making, to conduct education and training on the investigation and analysis methods and to determine a place for sharing information to reduce accidents. Universities are studying undisclosed, cutting-edge technologies for innovation. Accident investigation reports should focus solely on safety processes without exposing the state-of-the-art technology. When publishing, it is necessary to state details anonymously and describe only the processes related to the accident to ensure that personal information and experimental details of advanced technology are not exposed. Furthermore, as stipulated by the ICAO and the IMO^6,7^, the accident investigation report should not serve as grounds for determining the negligence rate and punishment of those involved in the accident but should be used to prevent accidents, which must be clarified.
