# Publisher Correction: A new approach for explosion accident prevention in chemical research laboratories at universities

**DOI:** 10.1038/s41598-022-08313-x

**Published:** 2022-03-10

**Authors:** Koji Fukuoka, Masao Furusho

**Affiliations:** 1 Risk Management Office, Kyushu University, 744 Motooka, Nishi-ku, Fukuoka, 819-0395 Japan; 2 President Office, National Institute of Technology, Oshima College, Oshima-gun, Japan

Correction to: *Scientific Reports* 10.1038/s41598-022-07099-2, published online 24 February 2022

This Article contained a typographical error in the Results section.

“The direct cause of both accidents Please check and confirm the edit and layis shown in Table 1, and the local workplace factors and organizational factors shown in Tables 2 and 3 have been clarified according to each SHEL element classification.”

now reads:

“The direct cause of both accidents is shown in Table 1, and the local workplace factors and organizational factors shown in Tables 2 and 3 have been clarified according to each SHEL element classification.”

The original Article has been corrected.

